# Extracorporeal Shock Wave Therapy with Low-Energy Flux Density Treatment Applied to Hemiplegia Patients on Somatosensory Functions and Spatiotemporal Parameters

**DOI:** 10.5152/eurasianjmed.2024.23270

**Published:** 2024-02-01

**Authors:** Gulnihal Deniz, Furkan Bilek, Arif Gulkesen, Murteza Cakir

**Affiliations:** 1 Department of Physiotherapy and Rehabilitation, Erzurum Technical University Faculty of Health Sciences, Erzurum, Turkey; 2 Department of Gerontology, Muğla Sıtkı Koçman University Fethiye Faculty of Health Sciences, Muğla, Turkey; 3 Department of Physical Medicine and Rehabilitation, Fırat University Faculty of Medicine, Elazığ, Turkey; 4 Department of Neurosurgery, Atatürk University Faculty of Medicine, Erzurum, Turkey; 5 Movement Disorders and Neuromodulation Center, Erzurum, Turkey

**Keywords:** Hemiplegia, spatiotemporal parameters, somatosensory, balance, spasticity, ESWT.

## Abstract

**Background:**

We aimed to investigate the effect of Extracorporeal Shock Wave Therapy (ESWT) applied to patients with hemiplegia on somatosensory data, spatiotemporal parameters, posture, and muscle tone.

**Methods:**

This was a double-blind, randomised, controlled trial. Patients were randomised within pairs to either the experimental (ESWT) group (n = 20) or the control group (n = 20). All patients participated in the same conventional stroke rehabilitation program for 60 minutes of treatment a day, 5 times a week for 6 weeks (30 sessions). Patients assigned to the ESWT group received additional ESWT over the plantar fascia 3 days/week for 6 weeks. Timed Up and Go (TUG) test, Modified Ashworth Scale (MAS) score, Posture Assessment Scale for Stroke Patients (PASS), spatiotemporal parameters, Semmes–Weinstein monofilament (SWM) test, and vibration sensation test (VST) were performed in all participant before and after treatment.

**Results:**

In the ESWT and control groups, statistically, significant differences were obtained in the posttreatment analysis than pre-treatment. Significant differences were found in foot angle, step cycle duration, swing phase, cadence, gait cycle distance, and VST values after ESWT treatment (*P* < .01).

**Conclusion:**

When combined with a neurological rehabilitation program, it was determined that ESWT applied to the plantar face of the foot in individuals with hemiplegia increased somatosensory functions and was more successful in developing postural control and balance.

**Main Points**

- Extracorporeal shock wave therapy was applied to the plantar area of the feet for the first time in hemiplegia.
- Extracorporeal shock wave therapy applied to the plantar area of the foot improved somatosensory sensation.
- Extracorporeal shock wave therapy applied to the plantar area of the foot improves gait, postural control, and balance.

## Introduction

Hemiplegia is a focal or global neurological deficit that develops suddenly due to disruption of cerebral blood flow due to vascular causes, lasting longer than 24 hours, or may result in death.^1^ Although it ranks first among the diseases that cause disability in adults, it causes fundamental health problems due to long-term disability.^2^ In individuals with hemiplegia, somatosensory function losses are also observed, along with motor loss. Although somatosensory disorders are dominant in approximately half of the patients with hemiplegia, they are often overlooked in clinics and research on hemiplegia.^3^ In the literature, somatosensory functions are critical for motor performance, sensory feedback, and learning through central processing. Loss of somatosensory function often leads to learned disuse and further functional impairment. Research findings have demonstrated that rehabilitation strategies tailored to specific sensory modalities can enhance the restoration of motor function. It is essential to evaluate the senses and motor functions.^3,4^ Gait disturbance is a common problem after hemiplegia, and despite intensive physiotherapy programs, many patients continue their lives with permanent gait problems. Gait disturbances primarily stem from sensorimotor dysfunction, which manifests as muscle weakness, perceptual and proprioceptive impairments, spasticity, or hypotonia.^5^ Improvement in gait is one of the functional goals after hemiplegia. However, only 60%-70% of patients can achieve this goal. While it is evident that the initial priority in post-hemiplegia treatment is the restoration of walking ability, the efficacy of various frequently employed physiotherapy modalities for enhancing gait remains less conspicuous. Extracorporeal shock wave therapy (ESWT) has emerged as a prospective therapeutic intervention in addressing spasticity following hemiplegia.^6,7^ The ESWT is used for various tendinopathies and other musculoskeletal disorders.^8^ Despite being a noninvasive therapeutic approach, ESWT remains a relatively uncharted domain. Further investigations are warranted to enhance comprehension of the intricate biological and medical repercussions associated with this treatment.^9^ Diverse methodologies, with a particular emphasis on spatiotemporal anatomical gait parameters encompassing cadence, gait cycle distance, maximum plantar pressure, step cycle duration, single-support phase, swing phase, step length, and foot angle, serve as tools to delineate the characteristics and extent of gait abnormalities in patients. These techniques are instrumental in assessing the efficacy of rehabilitation interventions following hemiplegia.^10^

Our study aims to investigate the effect of ESWT with low-energy flux density applied to the soles of the foot of hemiplegic patients on muscle tone, posture, and spatiotemporal parameters.

## Material and Methods

**Figure 1.**
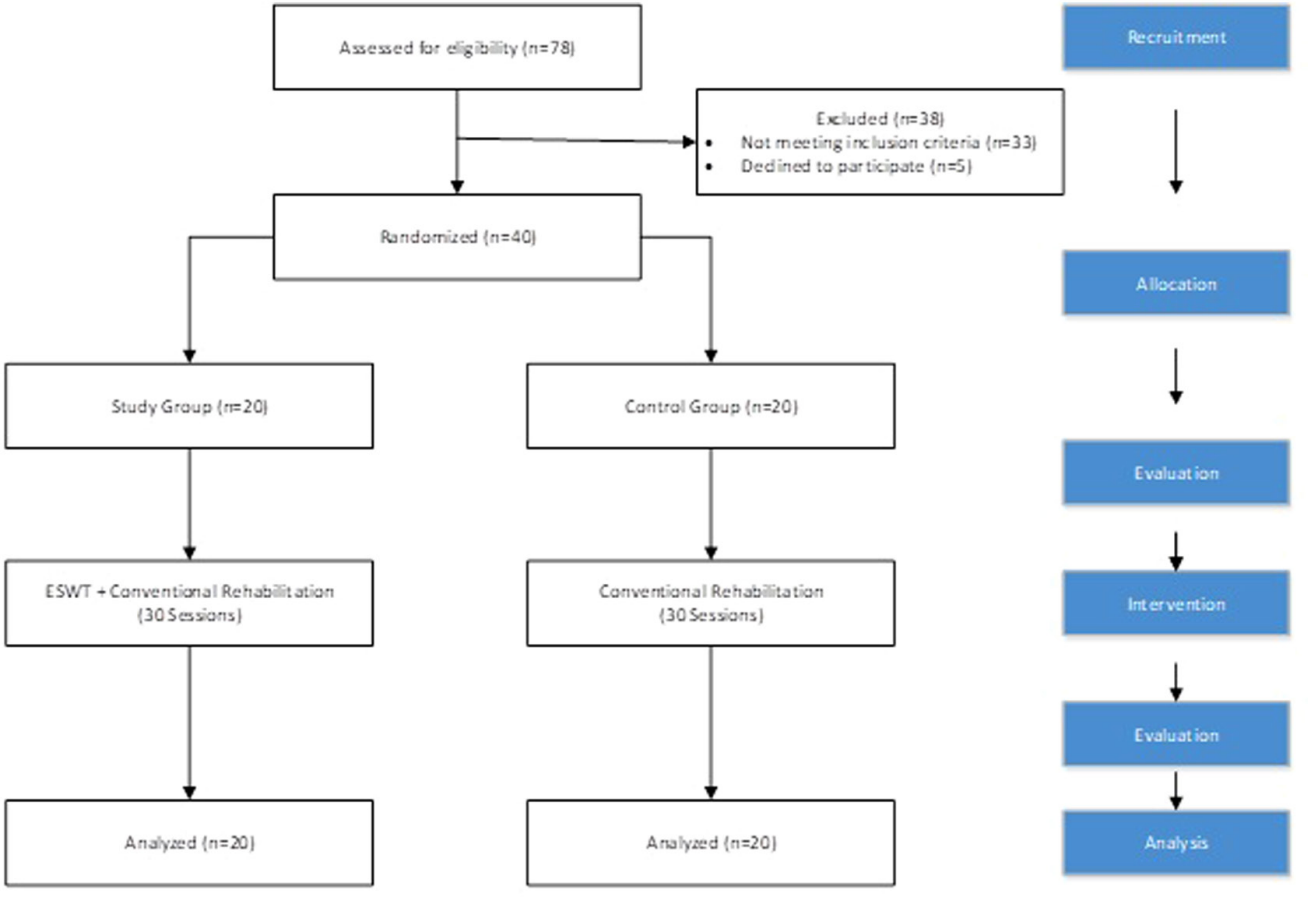
Flowchart of study.

The design of this study followed a double-blind, randomized controlled trial methodology. To ensure blinding, patients remained unaware of the nature of the intervention, and the evaluator responsible for assessments remained blinded to the group assignments throughout the study. The interventions were performed by 2PhD-level physiotherapists working in the field of neurological rehabilitation with at least ten years of experience in the use of ESWT. The assignment of patients to either the experimental group (ESWT) with a sample size of 20 or the control group with an equal sample size of 20 was accomplished using a randomized computerized allocation procedure (Figure 1).

### Participants

All participants were provided with comprehensive verbal and written details regarding the study. Prior to their involvement in the study, written informed consent was obtained from each participant. The study comprised a cohort of forty patients who had undergone standard physiotherapy and rehabilitation services at University Hospital within the timeframe spanning from July 2021 to January 2022. Detailed demographic and clinical profiles, including but not limited to gender, age, hemiplegia laterality, diagnostic history, body weight, height, dominant hand, body mass index (BMI), stroke etiology, poststroke duration, family medical background, individual medical history, patient medical records, prescribed medications, usage of assistive devices, as well as occupational and educational status, were thoroughly assessed for each participant. Both study groups participated actively in a conventional rehabilitation program, with patients assigned to the ESWT group receiving supplementary ESWT.

The study’s inclusion criteria encompassed individuals experiencing their initial onset of hemiplegia, with a minimum hemiplegia duration of 6 months, and presenting with stable spasticity within the triceps surae muscle group, characterized by a minimum muscle tone grade of 1 on the Modified Ashworth Scale (MAS). Furthermore, eligible participants were required to possess the capability to ambulate independently, either with or without the use of an orthosis, and to demonstrate proficiency in comprehending and adhering to verbal instructions.

The study’s exclusion criteria comprised individuals presenting with ataxia, dystonia, or dyskinesia, as well as those diagnosed with sensory impairments or dementia. Additionally, patients with bilateral hemiplegia were excluded from participation. Furthermore, individuals demonstrating a reduction in the range of motion (ROM) of the affected ankle dorsiflexion exceeding 10% compared to the unaffected side were ineligible for inclusion. Patients currently undergoing treatment with antispastic medications or actively receiving therapy for spasticity were also excluded from the study.

### Compliance with Ethical Standards

Ethical approval for our study was approved by the Ethics Committee of the Fırat University (decision number: 2021/08-14; date: June 24, 2021). According to the Declaration of Helsinki, all patients gave written informed consent to be enrolled in the study.

### Study Design

In line with the literature, range of motion exercises are also included in the conventional treatment program applied to patients with indications in the hospital hemiplegic patient treatment protocol.^11-13^

Conventional treatment aims to maintain joint flexibility and prevent contractures. For this, ROM exercises are started in the early stages. In addition, it includes stretching exercises, exercises that improve balance and mobility, and exercises that increase daily life activities. As the patient’s motor functions improve, active strengthening exercises for treating the hemiplegic extremity and techniques to increase coordination and dexterity are added.

Each patient in the study adhered to a standardized conventional stroke rehabilitation program, which entailed 60-minute daily treatment sessions delivered 5 days a week and spanning 6 weeks, encompassing 30 treatment sessions. In addition to this rehabilitation program, patients assigned to the ESWT group received supplemental ESWT sessions specifically targeting the plantar fascia. These additional ESWT sessions were administered 3 times a week over the same 6-week period.

The conventional therapy programs were individually tailored to each patient’s needs and predominantly centered around physiotherapeutic interventions. Regarding the hemiparetic side, therapeutic interventions encompassing proprioceptive neuromuscular facilitation techniques and range-of-motion exercises were conducted daily for 20 minutes. This regimen was administered 5 times weekly over 6 weeks, totaling 30 sessions. Passive mobilization and stretching exercises were applied for 15 minutes of therapy daily, 5 times a week for 6 weeks (30 sessions). Postural control exercises and balance training were implemented as part of the treatment regimen for 15 minutes each day. This therapeutic regimen was carried out 5 times a week for six weeks, comprising 30 sessions. Additionally, occupational therapy interventions were administered daily for 10 minutes, 5 days a week, over 6 weeks, resulting in 30 sessions.^2,14^ The basis of our occupational therapy is based on the active role of patients in their recovery processes by participating in functional activities. Functional tasks used in therapy include weight bearing of the upper extremity for postural support, reaching, carrying, lifting, grasping activities, and manipulating frequently used objects. 

Timed Up and Go (TUG) Test, MAS score, Posture Assessment Scale for Stroke Patients (PASS), spatiotemporal parameters, Semmes Weinstein Monofilament (SWM) Test, and Vibration Sensation Test (VST) were performed on all participants before and after treatment.

### Extracorporeal Shock Wave Therapy Protocol

The ESWT was applied with a 35 mm radial applicator in low-energy flux density (<0.08 Jm/mm^2^, 1000 shots) 3 days/week for 6 weeks to the plantar fascia by the same physiotherapist using the Modus Focused ESWT Device (İnceler Medikal, Ankara, Turkey).^15^

### Spatiotemporal Parameters

Spatiotemporal parameters play a crucial role in the analysis of gait, and their assessment was conducted using the Win-Track platform manufactured by MEDICAPTEURS Technology, based in France. This platform was employed to capture both plantar pressure patterns and gait parameters during unshod walking.

The Win-Track platform possesses physical dimensions of 1610 mm in length, 652mm in width, and 30mm in height, with a thickness of 9mm. This advanced platform fulfills a dual role in the assessment of both a patient’s stationary postural alignment and the dynamic parameters associated with their gait. Data generated by the platform was transferred to a computer equipped with automated algorithms for step identification and subsequent parameter calculations.

**Figure 2.**
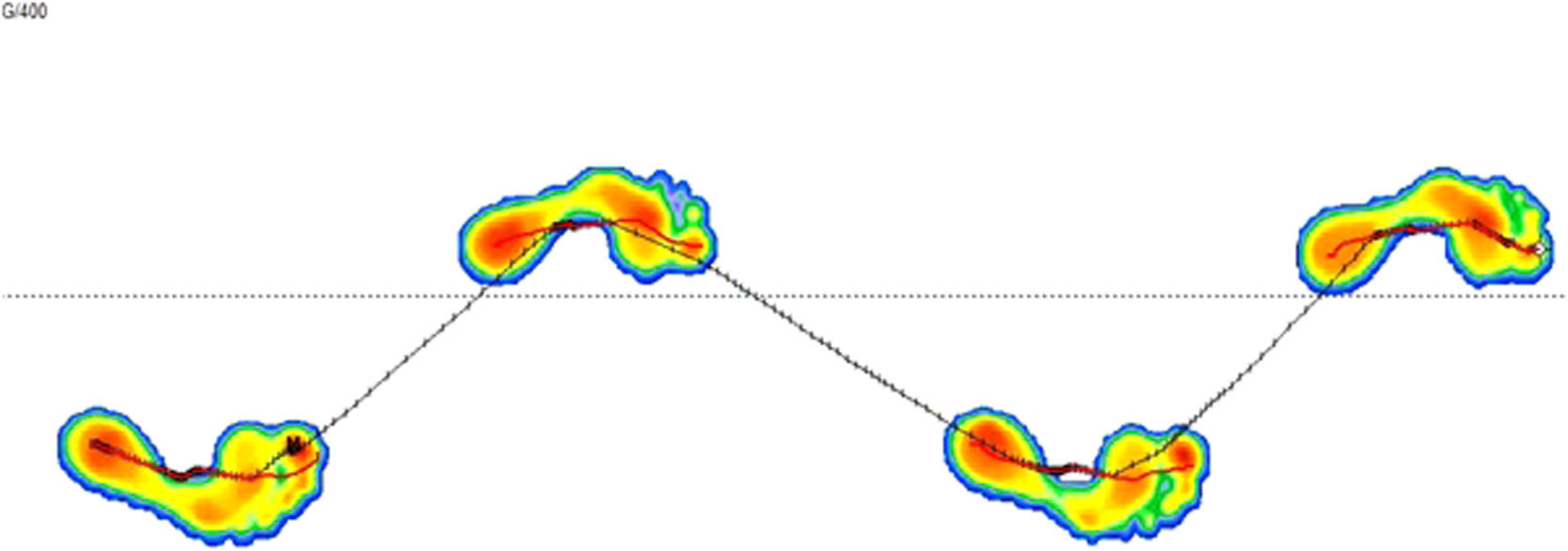
Evaluation of spatiotemporal parameters.

During the assessment, each participant was instructed to undertake 3 separate attempts, ensuring that a minimum of 3 strides were recorded while individuals traversed the platform. Exhibitors were specifically guided to maintain a forward gaze and walk comfortably on the platform to minimize any directional bias. Furthermore, weight distribution data was recorded while participants maintained a static stance on the platform^16^ (Figure 2).

### Timed Up and Go

The test involves a sequence of movements wherein individuals rise from a seated position in a chair, traverse a distance of 3 meters, execute a 180° turn, return to the chair, and resume a seated role. To ensure uniformity, the initial posture of participants was standardized, with their feet resting flat on the floor and their arms positioned on the chair’s armrests. Participants were instructed to complete this test at their own comfortable pace while striving to execute it as swiftly as possible. The test procedure commenced with the verbal command “start” and concluded when the individual successfully regained a seated position on the chair.^17^

### Modified Ashworth Scale Score

The evaluation of spasticity in both the upper and lower extremities was conducted employing the MAS before and after the treatment regimen. The MAS scale rates spasticity levels on a scale ranging from 0, indicating the absence of any increase in muscle tone, to 4, signifying a state of rigidity in the affected part, either in flexion or extension.^18^

### The Posture Assessment Scale for Stroke Patients

PASS was specifically designed for the assessment of postural control, and balance among stroke patients. This scale encompasses a set of 12 items that progressively increase in complexity. Utilizing a 4-point rating system, with “0” representing the lowest score and “3” indicating the highest, the total scoring potential on this scale ranges from 0 to 36.^19^

### Semmes Weinstein Monofilament

The assessment of the light touch pressure threshold on the plantar surface of the participants’ feet was conducted using a 5-piece monofilament (2.83/3.61/4.31/4.56/6.65 g) set of Chattanooga.^20,21^

### Vibration Sensation Test

The assessment of vibration sensation duration was conducted employing a 128-Hz frequency tuning fork (Elcon® Medical Instruments, Tuttlingen, Germany) placed at the first metatarsal head. The measurement of vibration duration was performed with a chronometer, commencing when the tuning fork made contact with the participant’s skin and concluding when the subject verbally indicated, “it has finished.” The recorded result was the average of 3 repeated trials, expressed in seconds.^22,23^

### Statistical Analysis

Statistical analyses were carried out using SPSS®, version 22.0 (IBM SPSS Corp.; Armonk, NY, USA), developed by IBM Corporation, Armonk, NY, USA. In order to assess the appropriateness of the sample size, a post hoc power analysis was performed utilizing the G-Power 3.1.9.4 software tool. The analysis indicated a high effect size of 0.5 and a power of 0.86 at the 95% CI, with a significance level set at 0.05. These results affirm that the sample size aligns with the desired level of statistical power.^24^ Data were expressed as the mean values accompanied by their respective standard deviations. To assess distinctions between pre- and postintervention measurements, the Wilcoxon test was employed. Furthermore, the Mann–Whitney *U*-test scrutinized differences between the 2 groups.

## Results

In our study, we evaluated 40 individuals, 10 female (50%) and 10 male (50%), in both groups. The ESWT group consisted of 30% retired, 50% homemakers, and 15% self-employed, while the control group consisted of 70% retired and 30% housewives (*P *> .05). In our study, it was observed that the right side was the dominant extremity in both groups with a high rate (85%, 75%), and the right side was highly prevalent in the hemiplegic extremities (55%, 90%) (*P *> .05). In addition, the data covering demographic, personal, and disease-related characteristics of the participants in the ESWT and control groups before and after the study were found to be statistically similar (*P *> .05) (Table 1).

All analyses performed before and after treatment in the ESWT and control groups were also measured in the hemiplegic and entire extremities. In intragroup comparisons before and after treatment, significant differences were found in spatiotemporal parameters, especially in total weight transfer, maximum plantar pressure, foot angle, step cycle duration, swing phase, step length, cadence, and gait cycle distance (*P *< .05), (Table 2). In pre- and post-treatment, SWM and VST values were compared in the ESWT group; significant differences were found (*P *< .01), while significant differences were found only in the VST values in the control group (Table 2).

When comparing the 2 groups, it became evident that there were statistically significant differences in the post-treatment data analysis for both the ESWT and control groups compared to the pretreatment data (Table 3). After ESWT treatment, statistically more significant differences were found in spatiotemporal and somatosensory parameters (*P *< .01).

## Discussion

Extracorporeal shock wave therapy is an easy and effective application of shock wave technology developed recently. It uses a pneumatic rocket mechanism to generate pressure waves in order to obtain radial waves. The rocket mechanism transmits accelerated compressed air to the treatment head. Thus, the kinetic energy is converted into a shock wave. Throughout the treatment process, this head remains in direct contact with the patient’s skin, thereby transmitting pressure waves to both the skin and the underlying subcutaneous tissues. Since it does not focus on a single point, it is easily used to treat large body areas. Especially in recent years, it has been seen in the literature that it is applied to different regions in patients with hemiplegia.^6,25^

In our study, we provided somatosensory input and reduced the tension in the plantar fascia by applying it to the plantar surface of the foot. Thus, the therapeutic effect of ESWT on somatosensory data, gait parameters, posture, and muscle tone has emerged. For this reason, it is appropriate to add ESWT to the conventional treatment program.

Extracorporeal shock wave therapy has been added to the rehabilitation programs of patients with hemiplegia and its effectiveness has been proven. However, the long-term efficacy, especially for the lower extremity, has yet to be thoroughly evaluated.^7^ The literature examined the effectiveness of ESWT treatment applied to patients with hemiplegia, mostly on spastic muscle.^26-29^ In addition to these, in our study, we examined the effect of ESWT application on the plantar surface of the foot on somatosensory parameters, balance, sensation, and spatiotemporal parameters in individuals with hemiplegia. To the best of our knowledge, our study represents the inaugural investigation of its kind in which ESWT is applied to the plantar surface of the foot of hemiplegic patients, and its effects on somatosensory parameters, sensory, and spatiotemporal parameters are examined.

The fact that the age, BMI, MAS, PASS, and TUG parameters of the hemiplegic individuals in both groups participating in our study were similar proves that our study data was balanced and consistent (*P *> .05). All parameters were evaluated before and after treatment within and between groups.

In our study, noteworthy statistically significant discrepancies were observed in the MAS, PASS, and TUG scores when comparing their respective values before and after the treatment, all within the same group. This showed that the rehabilitation program applied in both the control and ESWT groups was influential in treating spasticity. In addition, no statistically significant differences were found in the MAS scores between the groups (*P *> .05). Lee et al,^28^ Radinmehr et al,^24^ Wu et al,^30^ and Tirbisch^31^ found no significant difference in MAS scores in ESWT and control groups in their studies on hemiplegia patients, similar to our research. Taheri et al^26^ argued that ESWT applied to hemiplegic patients significantly reduced spasticity compared to the control group.

The validity of PASS and TUG has been accepted in the literature to evaluate functional balance in patients with hemiplegia.^32-34^ However, in the literature, spasticity assessment was primarily performed in hemiplegia patients who underwent ESWT, and balance and somatosensory parameters were not evaluated.^35^ Radinmehr et al^24^ found no significant difference in TUG scores in patients with hemiplegia treated with ESWT. Bilek and Tekin^36^ did not see a statistically significant difference in TUG scores between groups. Our study found statistically significant differences in the TUGs before and after the treatment. However, it is worth noting that no statistically significant distinction was detected between the groups. This showed that both treatments in the groups were effective in hemiplegia, and the balanced development was similar.

Hemiplegic individuals often exhibit a diverse array of gait disturbances characterized by aberrant kinematic gait patterns, spatiotemporal disparities, and somatosensory impairments that manifest in both the affected and unaffected lower extremities.^37^ Asymmetrical weight distribution in the extremities, coupled with disruptions in both the stance and swing phases of gait, reduces cadence and walking velocity among hemiplegic individuals. Additionally, diminished proprioceptive awareness of foot position compromises locomotor efficiency and presents a significant impediment to the patient’s prospects for functional recovery. The sense of the feet sole is important in protecting the support surface and creating appropriate motor responses according to the stimuli from the ground. A total of 70% of the receptors that provide sole foot sensation are fast-adapting receptors. Receptors provide appropriate weight transfer and balance control with high-speed adaptation ability.^38^ Research has revealed that, beyond the motor deficits observed in hemiplegic patients, somatosensory impairments in the lower extremities also exert influence on aspects such as postural control, precise foot placement, and the occurrence of errors during obstacle avoidance performance.^37^ Therefore, clinical studies emphasize the importance of somatosensory feedback in hemiplegia rehabilitation and argue that sensory functions should be evaluated and treated and motor functions improve gait phases.^39^ Gorst et al^37^ calculated that 56% of the hemiplegic patients were affected by somatosensory disorders and stated that evaluating lower extremity somatosensory disorders after hemiplegia and developing a treatment program can minimize disability and reduce falls. Kessner et al^4^ argued that somatosensory disorders constitute 50%-80% of hemiplegia patients. Although there is a continuous increase in hemiplegia patients, somatosensory evaluation and treatment are superficial and inadequate.^4^ In our study, we aimed to activate somatosensory functions by providing sensory input by applying ESWT to the plantar surface of the foot. Since the mean SVM test for light touch and pressure sense analysis in both groups before treatment was between 4.56 and 6.65, it was clinically classified as “protective sensory loss.” Since the mean SWM test in hemiplegics who underwent ESWT after treatment is between 3.84 and 4.31, they are in the “decreased protective sensation” class^38-40^ (Table 2). Once again, our study identified a notable disparity between the groups in the SWM test results following the treatment (*P *< .001). The VST test performed before the treatment to analyze vibration sense in both groups is in the “decreased” sensory class since the averages were between 1 and 9. Since the mean of the VST test is >10 in hemiplegia who underwent ESWT after treatment, it is in the “normal sense” class.^38-40^ Following the treatment, a noteworthy divergence was evident between the groups in the VST results (*P *< .001). Our study showed that ESWT applied to the plantar surface of the foot can be an effective treatment for the inputs of light touch, pressure sense, and vibration sense in patients with hemiplegia. After hemiplegia, 75% of individuals experience gait disorders. Hemiparetic patients have a typical gait pattern with the affected upper extremity flexion and lower extremity extension, with abduction and lower extremity rotation (sickling).^41^ The gait pattern observed in hemiparetic patients combines existing deficiencies and compensation mechanisms against them. In hemiparetic patients, problems include insufficient shock absorption during heel strike during walking, insufficient momentum control in the stance phase, inability to create enough force to take a forward step, and inability to advance the paralyzed extremity quickly enough in the swing phase have been observed. Spatiotemporal parameters are evaluated in gait problems in hemiparetic patients.^42-44^ Speed is associated with both temporal and spatial characteristics of gait.^16^ Shin et al^45^ observed an improvement in cadence and walking speed, a reduction in the swing phase duration, and decreased bilateral asymmetry in extremity movements following treatment. Conversely, findings from Chen et al^46^ suggested that the hemiplegic side exhibited higher values in terms of swing phase duration and step length. The observed gait asymmetry in their study was attributed to a disorder in the initiation of the swing phase in the hemiplegic extremity. Their research posited that spatiotemporal parameters, encompassing factors like step length, swing phase duration, and stance time, exert a substantial influence in shaping the distinctive characteristics of gait. In the literature, sensorial and spatiotemporal parameters have not been evaluated in patients with hemiplegia undergoing ESWT. In addition, in the literature, ESWT treatment was mostly applied to spastic muscle patients diagnosed with hemiplegia.^ 26-29^ Our study is the first study in which somatosensory input was applied to the plantar surface of the foot, not the spastic muscle. Total weight transfer, forefoot pressure analysis, hindfoot pressure analysis, and maximum plant and r pressure decreased; significant differences were found in both groups, mostly in the ESWT group (*P *< .01). In comparing the groups, significant differences were found in total weight transfer and hindfoot pressure analysis after treatment. In the ESWT group, the difference between the affected and unaffected extremities decreased more than in the control group after treatment. In both study groups, higher foot angle values were consistently observed on the hemiparetic side. This phenomenon suggests that the body employs a compensatory mechanism by broadening the support surface to preserve balance. Furthermore, both groups exhibited elevated measurements for step cycle duration, swing phase duration, and step length on the hemiparetic side. After treatment, a noteworthy reduction in these parameters was documented in both groups, with statistically significant differences noted when compared to pretreatment values. Additionally, in response to treatment, both groups demonstrated significant increases in cadence and gait cycle distance values compared to their respective pretreatment states.

In conjunction with a neurological rehabilitation program for hemiplegic patients, the application of ESWT to the plantar aspect of the foot has been shown to enhance somatosensory functions and lead to notable improvements in gait, postural control, and balance. While neurological rehabilitation interventions in isolation are effective in mobilizing hemiplegic patients, the assessment of spatiotemporal parameters remains crucial to ascertain the treatment’s efficacy and its impact on patient outcomes.

Our study findings substantiate that the treatment administered to hemiplegic patients yields significant enhancements in their functional status, marked by a substantial reduction in gait abnormalities. Notably, in contrast to existing literature, our results suggest that ESWT targeting the plantar region of the feet has the potential to augment somatosensory functions, particularly when employed in conjunction with conventional physical therapy. This combined approach appears to yield superior outcomes in terms of gait improvement, postural control, and balance for hemiplegic individuals.

An inherent strength of our study lies in its utilization of a double-blind, randomized, controlled trial design. Our investigation involved a comparative analysis, pitting ESWT in conjunction with neurological rehabilitation against neurological rehabilitation alone as distinct rehabilitation modalities for patients afflicted with hemiplegia.

The limitation of our study is the small number of subjects. Another is that more tests should be added to sensory assessment methods.

## Figures and Tables

**Table 1.** Demographic and Disease-related Features of the Groups

	ESWT Group Mean ± SD	ESWT Group (Minimum–Maximum)	Control Group Mean ± SD	Control Group (Minimum–Maximum)	*P*
Age (years)	61.05 ± 14.01	20-88	59.50 ± 6.94	49-75	.41
BMI (BT) (kg/m^2^)	29.05 ± 4.46	20.51-36.36	28.53 ± 4.27	20.51-33.64	.62
BMI (AT) (kg/m^2^)	28.99 ± 4.38	21.06-36.85	28.18 ± 4.20	21.10-33.49	.41
TUG (BT) (ms)	20.82 ± 7.45	10.13-38.13	21.26 ± 7.25	10.13-38.13	.84
TUG (AT) (ms)	17.90 ± 6.51	8.58-34.68	19.50 ± 6.58	9.25-28.58	.31
MAS (BT) upper ext	1.80 ± 0.86	1-4	1.5 ± 0.53	1-3	.38
MAS (AT) upper ext	1.40 ± 0.61	1-3	1.2 ± 0.41	1 ± 2.5	.36
MAS (BT) lower ext	1.87 ± 0.60	1-3	1.5 ± 0.44	1-2.5	.07
MAS (AT) lower ext	1.25 ± 0.41	0.5-2	1.2 ± 0.29	1-2	.79
PASS (BT)	18.20 ± 5.26	12-32	20.65 ± 5.11	11-29	.16
PASS (AT)	26.30 ± 4.96	18-34	24.90 ± 4.91	15-32	.38

AT, after treatment; BMI, body mass index; BT, before treatment; ESWT, extracorporeal shock wave therapy; ext, extremity; MAS, Modified Ashworth Scale; PASS, Posture Assessment Scale for Stroke Patients; TUG, timed up and go test.

**Table 2.** Comparison of Spatiotemporal, Balance, and Somatosensory Values of Patients Pre-treatment and Post Treatment

	Group	Side	Mean ± SD		*P*	Δ = Pre-treatment −Post Treatment	Δ*P*
			Pre-treatment	Post Treatment			
Total weight transfer % (SS)	ESWT	Affected	43.05 ± 9.96	48.25 ± 3.55	.019*3	−5.20 ± 9.32	.094
		Unaffected	56.95 ± 9.96	51.75 ± 3.55	.019*3	5.20 ± 9.32	
	Control	Affected	43 ± 3.94	44.5 ± 3.06	.015*3	−1.50 ± 2.43	.168
		Unaffected	55.55 ± 5.83	54.05 ± 3.80	.246	1.50 ± 7.19	
Forefoot pressure analysis % (SS)	ESWT	Affected	19.05 ± 8.01	22.10 ± 2.19	.176	−3.05 ± 7.30	.608
		Unaffected	23.15 ± 7.26	24.25 ± 3.90	.285	−1.10 ± 8.37	
	Control	Affected	18.65 ± 2.25	20.85 ± 2.20	.001**3	−2.20 ± 0.69	.855
		Unaffected	23.15 ± 4.73	23.85 ± 1.72	.512	−0.70 ± 4.97	
Hindfoot pressure analysis % (SS)	ESWT	Affected	24.15 ± 6.41	26.10 ± 2.75	.121	−1.95 ± 5.39	.282
		Unaffected	33.85 ± 6.50	27.65 ± 4.22	.003**3	6.20 ± 6.93	
	Control	Affected	24.40 ± 2.89	24.65 ± 5.05	.388	−0.25 ± 4.41	.058
		Unaffected	32.45 ± 5.06	30.20 ± 3.25	.099	2.25 ± 5.79	
Maximum plantar pressure (g/cm^2^)	ESWT	Affected	1312.60 ± 145.55	1430.15 ± 135.72	.006**3	−117.55 ± 135.36	.056
		Unaffected	1490.25 ± 277.65	1518.65 ± 171.19	.048*3	−28.40 ± 153.87	
	Control	Affected	1321.15 ± 105.84	1375.50 ± 99.08	.001**3	−54.35 ± 46.94	.599
		Unaffected	1457.20 ± 109.27	1504.55 ± 96.56	.001**3	−47.35 ± 43.55	
Foot angle (degrees)	ESWT	Affected	9.85 ± 5.55	5.94 ± 3.87	.001**3	3.90 ± 2.97	.0001**3
		Unaffected	4.34 ± 3.09	2.46 ± 2.04	.001**3	1.88 ± 2.07	
	Control	Affected	8.12 ± 3.14	7.06 ± 2.33	.001**3	1.05 ± 1.22	.038*3
		Unaffected	3.46 ± 1.68	2.66 ± 1.55	.001**3	0.79 ± 0.86	
Step cycle duration (ms)	ESWT	Affected	1057.45 ± 337.56	857.65 ± 291.54	.001**3	199.80 ± 219.64	.016*3
		Unaffected	903.30 ± 291.35	745.15 ± 249.19	.001**3	72.70 ± 47.67	
	Control	Affected	1303.80 ± 126.14	1231.10 ± 143.59	.001**3	158.15 ± 220.69	.085
		Unaffected	1069.55 ± 127.18	1000.90 ± 121.39	.001**3	68.65 ± 49.57	
Swing phase (ms)	ESWT	Affected	2549.20 ± 798.86	1976.30 ± 611.10	.001**3	572.90 ± 393.18	.001**3
		Unaffected	2300.50 ± ± 700.16	1727.50 ± 507.26	.001**3	213.00 ± 168.93	
	Control	Affected	2545.50 ± 786.64	2332.50 ± 733.06	.001**3	573.00 ± 353.79	.0001**3
		Unaffected	2204 ± 711.34	2035 ± 698.51	.001**3	169.00 ± 105.27	
Step length (mm)	ESWT	Affected	564.20 ± 255.98	480.95 ± 239.82	.004**3	83.25 ± 125.66	.868
		Unaffected	466.75 ± 250.33	418.25 ± 219.08	.021*3	48.50 ± 112.07	
	Control	Affected	631.30 ± 170.33	578.45 ± 157.97	.001**3	48.50 ± 112.07	.916
		Unaffected	580 ± 165.77	534.30 ± 154.05	.001**3	45.70 ± 35.92	
Cadence (number/min)	ESWT		68.13 ± 18.90	81.61 ± 19.02	.002**3	−13.48 ± 13.22	.024*3
	Control		64.41 ± 11.96	70.60 ± 13.50	.001**3	−6.19 ± 4.02	
Gait cycle distance (mm)	ESWT		793.80 ± 181.32	870.40 ± 177.50	.001**3	−76.60 ± 92.85	.021*3
	Control		651.50 ± 132.63	677.35 ± 137.75	.001**3	−25.85 ± 15.11	
SWM	ESWT		4.73 ± 0.85	4.06 ± 0.46	.001**3	0.67 ± 0.65	.293
	Control		4.95 ± 1.04	4.53 ± 0.57	.046	0.41 ± 0.85	
VST	ESWT		5.24 ± 1.656	11.69 ± 2.06	.001*3	−6.45 ± 2.11	.0001**3
	Control		6.15 ± 3.24	8.12 ± 2.52	.006**3	−1.97 ± 2.64	

“Unaffected” refers to the healthy side, while “Affected” refers to the hemiplegic side. Values in bold indicate statistical significance.

ESWT, extracorporeal shock wave therapy; SWM, Semmes–Weinstein monofilament test; VST, vibration sensation test.

*P < .05.

**P < .01.

**Table 3.** Comparison of Spatiotemporal, Balance, and Somatosensory Values of Patients Between Groups with ESWT and Control

	Group and Side	Pre-treatment *P*	Post Treatment *P*
Total weight transfer % (SS)	ESWT affected side Control affected side	.383	.001**3
	ESWT unaffected side Control unaffected side	.883	.060
Forefoot pressure analysis % (SS)	ESWT affected side Control affected side	.183	.114
	ESWT unaffected side Control unaffected side	.355	.738
Hindfoot pressure analysis % (SS)	ESWT affected side Control affected side	.925	.003**3
	ESWT unaffected side Control unaffected side	.398	.011*3
Maximum plantar pressure (g/cm^2^)	ESWT affected side Control affected side	.820	.183
	ESWT unaffected side Control unaffected side	.947	.862
Foot angle (degrees)	ESWT affected side Control affected side	.495	.157
	ESWT unaffected side Control unaffected side	.565	.314
Step cycle duration (ms)	ESWT affected side Control affected side	.023*3	.0001**3
	ESWT unaffected side Control unaffected side	.0001**3	.0001**3
Swing phase (ms)	ESWT affected side Control affected side	.925	.108
	ESWT unaffected side Control unaffected side	.620	.142
Step length (mm)	ESWT affected side Control affected side	.076	.035*3
	ESWT unaffected side Control unaffected side	.010*3	.011*3
Cadence (number/min)	ESWT	.565	.020*3
	Control		
Gait cycle distance (mm)	ESWT	.004**3	.001**3
	Control		
SWM	ESWT	.398	.001**3
	Control		
VST	ESWT	.414	.001**3
	Control		

Student’s *t*-test was applied. Values in bold indicate statistical significance.

SWM, Semmes–Weinstein monofilament test; VST, vibration sensation test.

*P < .05.

**P < .01.
